# Supplementary material for: Seminoma and Embryonal Carcinoma Footprints Identified by Analysis of Integrated Genome-Wide Epigenetic and Expression Profiles of Germ Cell Cancer Cell Lines
Source: PLoS One. 2014 Jun 2;9(6):e98330. doi: 10.1371/journal.pone.0098330 (PMC4041891; doi:10.1371/journal.pone.0098330)
Supplement: File S1 — ZIP file containing DMRforPairs output for significant regions. Please start from the html files. (ZIP) [file pone.0098330.s008.zip › figures/17431.pdf]

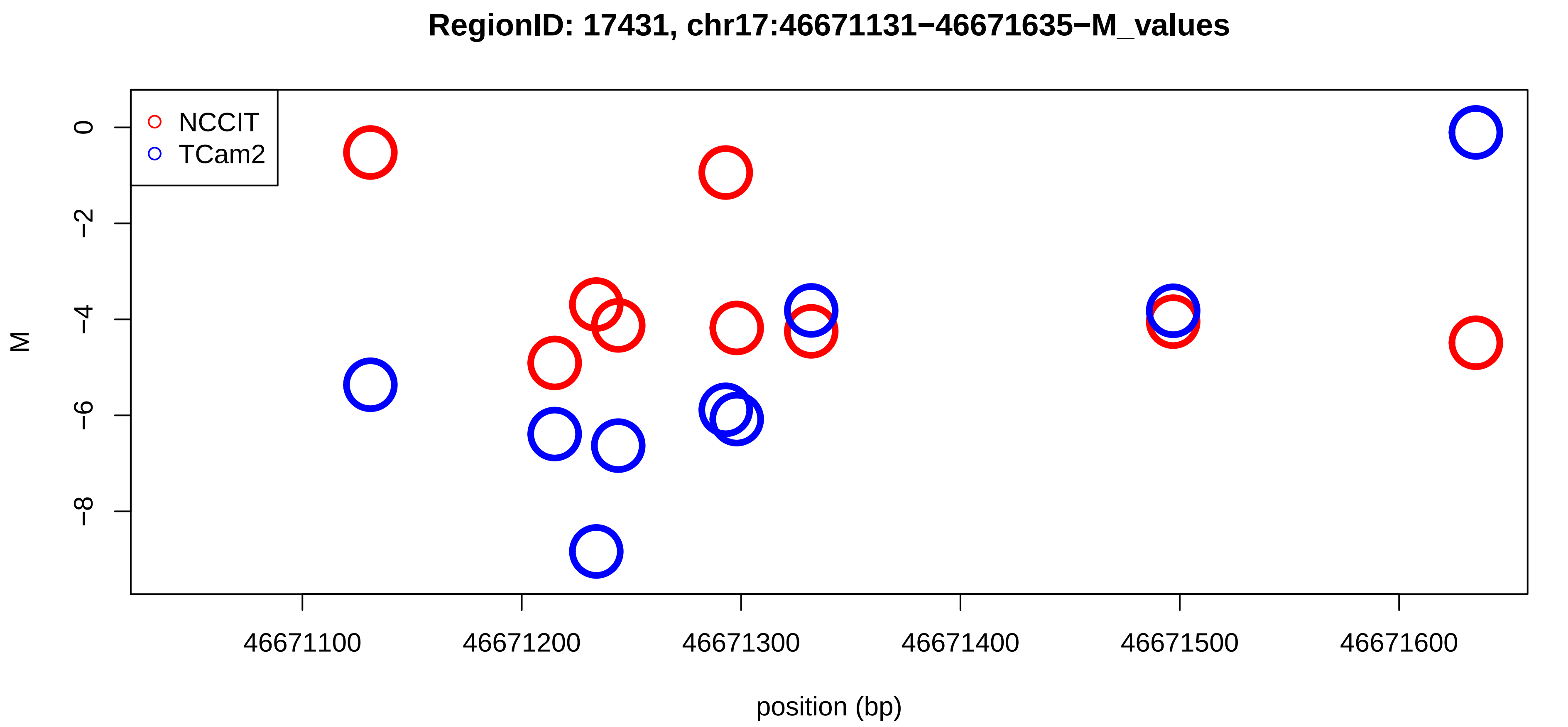
RegionID: 17431, chr17:46671131−46671635−M_values
NCCIT
TCam2
M
0
−2
−4
−6
−8
46671100
46671200
46671300
46671400
46671500
46671600
position (bp)

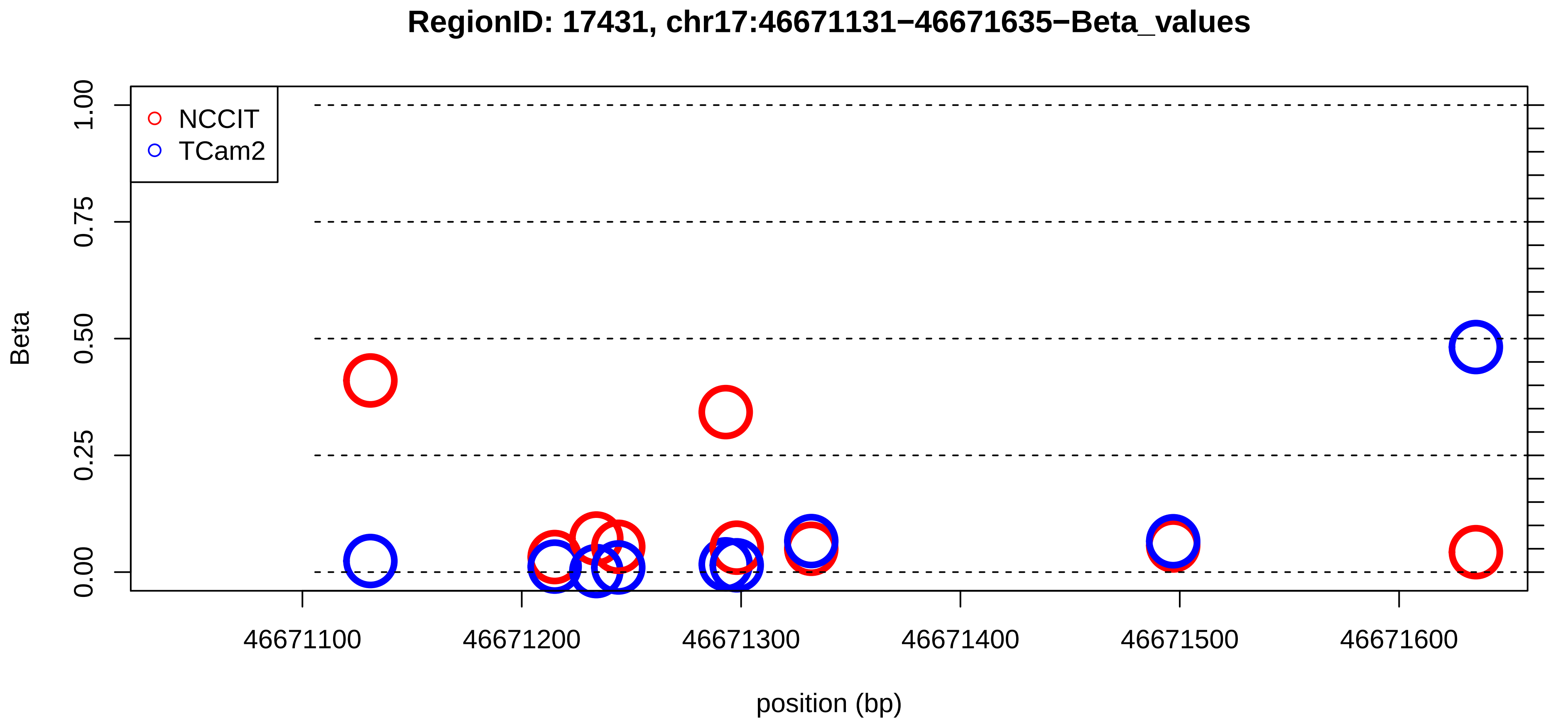
RegionID: 17431, chr17:46671131−46671635−Beta_values
NCCIT
TCam2
Beta
0.00
0.25
0.50
0.75
1.00
46671100
46671200
46671300
46671400
46671500
46671600
position (bp)
